# Supplementary material for: Genome Characterization of Nocturne116, Novel Lactococcus lactis-Infecting Phage Isolated from Moth
Source: Microorganisms. 2021 Jul 20;9(7):1540. doi: 10.3390/microorganisms9071540 (PMC8306868; doi:10.3390/microorganisms9071540)
Supplement: Supplementary file 1 [file microorganisms-09-01540-s001.zip › Supplementary_material_description.pdf]

Supplementary Materials: Figure S1: Neighbor-joining tree of 16S rRNA gene sequences of bacterial isolate LNT and closely-related bacterial species (*Bdellovibrio bacteriovorus* represents an outgroup). The evolutionary history was inferred using the Neighbor-joining method [1]. The optimal tree with the sum of branch length = 1199.79398267 is shown. The analysis involved 46 nucleotide sequences (16s rRNA gene sequences of isolate LNT, 44 other closely related bacterial species and *Bdellovibrio bacteriovorus* as an outgroup for rooting). Tip labels correspond to the taxa and are in the format of "Species|Strain|Accession". Isolate LNT is indicated by red font and clade corresponding to 16S rRNA sequences of *Lactococcus lactis* is highlighted in green. The percentage of replicate trees in which the associated taxa clustered together in the bootstrap test (1000 replicates) are indicated by the corresponding node color according to the legend [2]. The evolutionary distances were computed using the number of differences method [3] and are in the units of the number of base differences per sequence. The tree is drawn to scale, branch lengths correspond to the number of nucleotide changes and scale bar indicates 10 nucleotide differences. All positions with less than 90% site coverage were eliminated. That is, fewer than 10% alignment gaps, missing data, and ambiguous bases were allowed at any position. There were a total of 1385 positions in the final dataset. Evolutionary analyses were conducted in MEGA7 [4] and the resulting tree was visualized in FigTree v 1.4.4 [5]. **Figure S2**: Transmission electron micrograph of phage Nocturne116 stock negatively stained with 0.5% uranyl acetate. **Table S1**: Genome annotation and predicted functions of Nocturne116 ORFs. **Table S2:** Genome overviews of the currently publicly available *Lactococcus* phage complete genome entries (n=349). Taxonomy is provided as was retrieved from respective genome entries. Asterisk (*) after entry in "Host" column indicates that no resolution to particular species and/or strain was possible from genome entry metadata. "NA" entries in "VIRIDIC clustering (65% similarity as cluster threshold; 95% as species threshold; n=108)" column indicate that the respective genome was not a part of VIRIDIC analysis. Blanks in "vConTACT2 clustering (default settings; n=349)" "VC" column indicate that the proteome of the phage either overlapped multiple clusters or was a singleton or an outlier (seen in "VC.Status" column). **Figure S3**: Heatmap showing the results of VIRIDIC analysis of selected (elaborated on in main text) *Lactococcus* phages. **Figure S4**: Boxplots comparing genome sizes (**A**) and percentages of unannotated gene products (**B**) of complete genome entries belonging to the selected *Lactococcus* phage clusters as determined by nucleotide sequence similarity herein. *Skunavirus* and *Ceduovirus* entries were included based on the annotated complete genome entry associated taxonomical information. Some of the clusters correspond to the recently ratified novel *Lactococcus* phage genera (Viridic_cluster 14 ~ ICTV *Audreyjarvisvirus*; Viridic_cluster 3 ~ ICTV *Teubervirus;* Viridic_cluster 16 ~ ICTV *Vedamuthuvirus*, Viridic_cluster 17 ~ ICTV *Nevevirus*; Viridic_cluster 11 ~ ICTV *Fremauxvirues*). Figure generated using ggstatsplot R package [6].

Supplementary material references:

1. Saitou, N.; Nei, M. The neighbor-joining method: a new method for reconstructing phylogenetic trees. *Mol. Biol. Evol.* **1987**, *4*, 406–425, doi:10.1093/oxfordjournals.molbev.a040454.

2. Felsenstein, J. CONFIDENCE LIMITS ON PHYLOGENIES: AN APPROACH USING THE BOOTSTRAP. *Evolution* **1985**, *39*, 783–791, doi:10.1111/j.1558-5646.1985.tb00420.x.

3. Nei, M.; Kumar, S. *Molecular Evolution and Phylogenetics.*; Oxford University Press: New York, 2000;

4. Kumar, S.; Stecher, G.; Tamura, K. MEGA7: Molecular Evolutionary Genetics Analysis Version 7.0 for Bigger Datasets. *Mol. Biol. Evol.* **2016**, *33*, 1870–1874, doi:10.1093/molbev/msw054.

5. Rambaut, A. FigTree v. 1.4.4. *http://tree.bio.ed.ac.uk/software/figtree/* 2018.

6. Patil, I. Visualizations with statistical details: The "ggstatsplot" approach. *J. Open Source Softw.* **2021**, *6*, 3167, doi:10.21105/joss.03167.
