# Supplementary figures and images for: Genome Characterization of Nocturne116, Novel Lactococcus lactis-Infecting Phage Isolated from Moth
Source: Microorganisms. 2021 Jul 20;9(7):1540. doi: 10.3390/microorganisms9071540 (PMC8306868; doi:10.3390/microorganisms9071540)

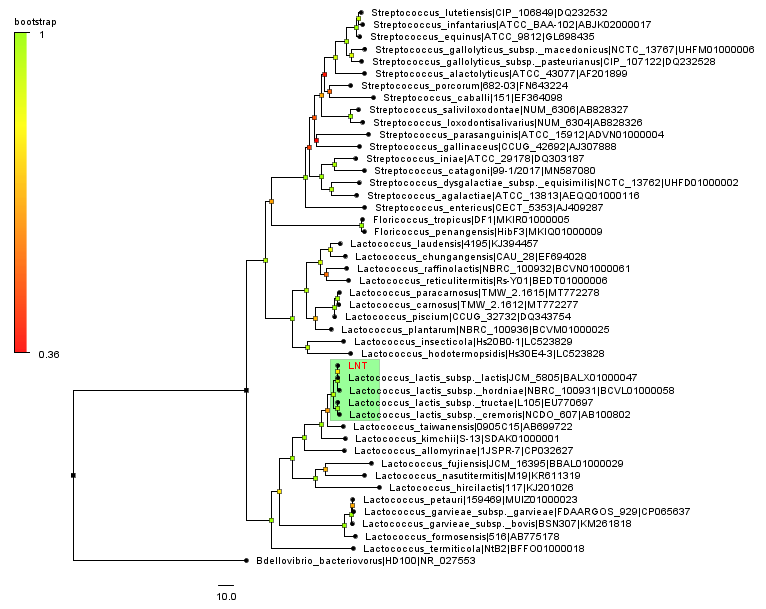

Supplement: Supplementary file 1 [file microorganisms-09-01540-s001.zip › Supplementary_figure_S1.png]

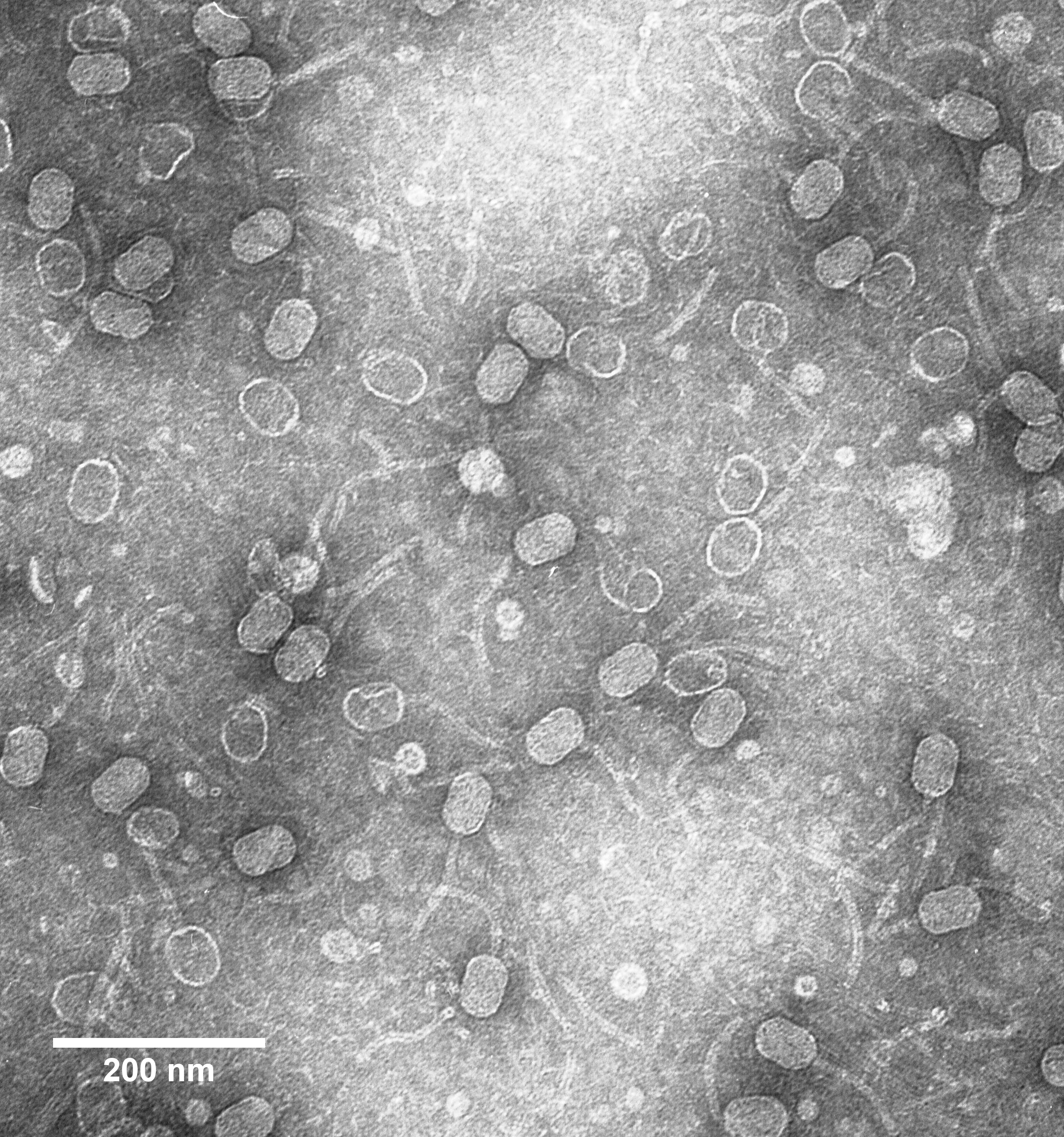

Supplement: Supplementary file 1 [file microorganisms-09-01540-s001.zip › Supplementary_figure_S2.tif]

# Aligned genome fraction

# Genome length ratio

0 0.25 0.5 0.75

Intergenomic distance

0 10 20 30 40 50 60 80 100

Genome length

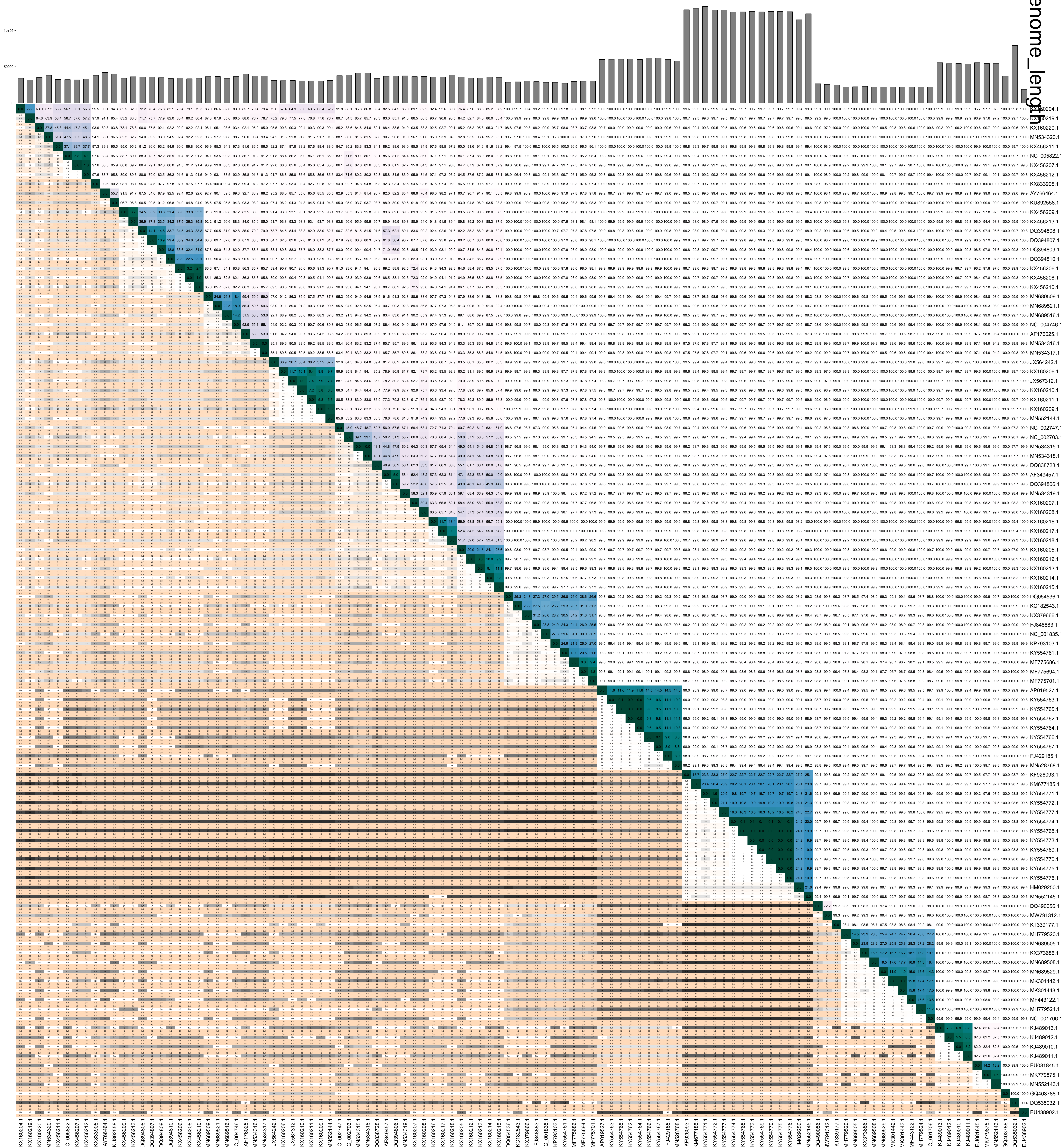

Supplement: Supplementary file 1 [file microorganisms-09-01540-s001.zip › Supplementary_figure_S3.PDF]

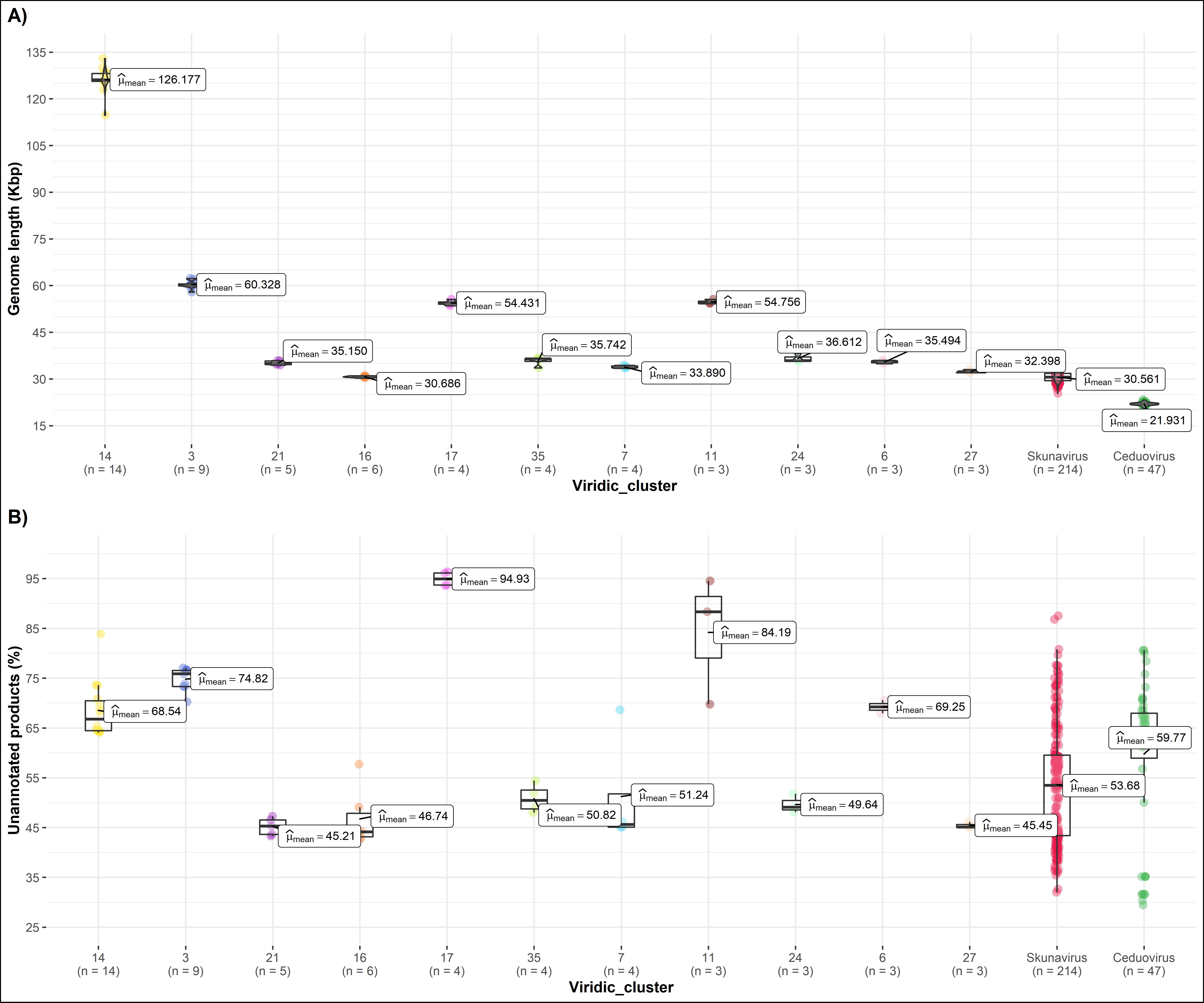

Supplement: Supplementary file 1 [file microorganisms-09-01540-s001.zip › Supplementary_figure_S4.jpg]
